# Reversal of Proximal Renal Tubular Dysfunction after Nucleotide Analogue Withdrawal in Chronic Hepatitis B

**DOI:** 10.1155/2017/4327385

**Published:** 2017-10-29

**Authors:** Abhasnee Sobhonslidsuk, Pawin Numthavaj, Jirachaya Wanichanuwat, Areepan Sophonsritsuk, Supanna Petraksa, Alongkorn Pugasub, Paisan Jittorntam, Anucha Kongsomgan, Sittiruk Roytrakul, Bunyong Phakdeekitcharoen

**Affiliations:** ^1^Department of Medicine, Division of Gastroenterology and Hepatology, Faculty of Medicine, Ramathibodi Hospital, Mahidol University, Bangkok, Thailand; ^2^Section for Clinical Epidemiology and Biostatistics, Faculty of Medicine, Ramathibodi Hospital, Mahidol University, Bangkok, Thailand; ^3^Department of Obstetrics and Gynecology, Faculty of Medicine, Ramathibodi Hospital, Mahidol University, Bangkok, Thailand; ^4^Office of Research Academic and Innovation, Faculty of Medicine, Ramathibodi Hospital, Mahidol University, Bangkok, Thailand; ^5^Department of Pathology, Faculty of Medicine, Ramathibodi Hospital, Mahidol University, Bangkok, Thailand; ^6^National Center for Genetic Engineering and Biotechnology, Pathumthani, Thailand; ^7^Department of Medicine, Division of Nephrology, Faculty of Medicine, Ramathibodi Hospital, Mahidol University, Bangkok, Thailand

## Abstract

**Aims:**

Proximal renal tubular dysfunction (PRTD) is an infrequent complication after nucleotide analogue therapy. We evaluated the outcomes of PRTD and nephrotoxicity after nucleotide analogue withdrawal in chronic hepatitis B (CHB).

**Methods:**

A longitudinal follow-up study was performed in patients with PRTD after nucleotide analogue discontinuation. Serum and urine were collected at baseline and every 3 months for one year. The fractional excretion of phosphate (PO_4_), uric acid (UA), and potassium and tubular maximal reabsorption rate of PO_4_ to glomerular filtration rate (TmPO_4_/GFR) were calculated. Renal losses were defined based on the criteria of substance losses. Subclinical PRTD and overt PRTD were diagnosed when 2 and ≥3 criteria were identified.

**Results:**

Eight subclinical and eight overt PRTD patients were enrolled. After nucleotide analogue withdrawal, there were overall improvements in GFR, serum PO_4_, and UA. Renal loss of PO_4_, UA, protein, and *β*2-microglobulin reduced over time. At one year, complete reversal of PRTD was seen in 13 patients (81.2%). Improvements in PRTD were seen in all but one patient.

**Conclusion:**

One year after nucleotide analogue withdrawal, PRTD was resolved in most patients. Changes in TmPO_4_/GFR, urinary protein, and *β*2-microglobulin indicate that urinary biomarkers may represent an early sign of PRTD recovery.

## 1. Introduction

Over 240 million people worldwide are chronically infected with hepatitis B virus (HBV), which can lead to cirrhosis and hepatocellular carcinoma [1]. The goal in chronic hepatitis B (CHB) treatment is to prevent the progression to end stage liver diseases and hepatocellular carcinoma. The ideal endpoint for the treatment of CHB is sustained off-therapy hepatitis B surface antigen loss [1–3]. If sustained off-therapy is not achievable, then a long-term virological suppression with oral antiviral drugs is preferred [1, 3]. Lamivudine (LAM) was the first oral antiviral drug approved for the treatment of CHB. However, long-term LAM therapy is associated with an emergence of LAM-resistant mutations, which require a modification in the treatment regimen by adding or switching nucleotide analogues including adefovir (ADV) or tenofovir (TDF) [1]. Currently, entecavir (ETV) and TDF are recommended as the preferred first-line oral antiviral therapy for CHB [1–3].

A slight reversible increase in serum creatinine is reported in fewer than 1% to 3% of patients after 3–5 years of treatment with ADV 10 mg and TDF 300 mg [4–6]. Despite the safety profile of 10 mg ADV from large clinical trials [4–6], ADV-induced Fanconi's syndrome, proximal renal tubular dysfunction (PRTD), and hypophosphatemia have been reported in CHB patients [7–13]. PRTD, hypophosphatemia, and Fanconi's syndrome are also common in human immunodeficiency virus- (HIV-) infected patients receiving TDF as a part of highly active antiretroviral therapy [14–16]. A recent meta-analysis study revealed no significant differences between ETV and TDF treatment in CHB patients in terms of renal safety profiles and hypophosphatemia; however, the short duration of the study limited the power of the conclusions [4–6, 17]. In contrast to these studies [4–6, 17], PRTD and impaired phosphate renal tubular reabsorption were seen in 15–48% of CHB patients receiving long-term ADV and TDF therapy as in real world clinical experience [8, 10, 18–20]. Reversibility of renal dysfunction has been shown in HIV-infected patients after cessation of TDF [21–25]. A few cases of reversible TDF or ADV-induced PRTD and Fanconi's syndrome have been reported in CHB patients after stopping treatment with nucleotide analogues [12, 26]. We aimed to prospectively assess the clinical and laboratory outcomes of PRTD in CHB patients after discontinuation of TDF or ADV.

## 2. Materials and Methods

### 2.1. Study Design and Patient Population

A prospective longitudinal study was performed at the liver clinics of Ramathibodi Hospital, Bangkok, Thailand, from 1 October 2014 to 30 September 2015. The study was approved by the Committee on Human Rights related to Research Involving Human Subjects, Faculty of Medicine, Ramathibodi Hospital (ID 11-56-29). The study was conducted in accordance with the Declaration of Helsinki (1964). Written inform consent was obtained from the study subjects before recruitment. Out of 92 CHB patients who had been treated with nucleotide analogue, 24 patients were found to have nucleotide analogue-related PRTD from our previous study [20]. Renal loss of protein, glucose, phosphate, uric acid, potassium, and bicarbonate was defined via laboratory assays. PRTD was diagnosed when at least two of these criteria were present [10, 16, 20, 27]. Subclinical PRTD was defined at two criteria present, and overt PRTD was defined with ≥3 criteria [20]. Seven patients with subclinical PRTD chose to continue ADV or TDF taking and have renal tubular function monitored periodically at the liver clinics. ADV or TDF was discontinued and replaced with ETV or LAM in 17 patients and all of them were invited to the study. Ten patients had history of LAM resistance and/or inadequate virological suppression from LAM monotherapy and required the addition of ADV or TDF. All of them had undetectable HBV viral load more than 3 years prior to enrollment. ADV and TDF were started as primary antiviral therapy in 7 patients.

We excluded CHB patients with LAM-resistant mutations who needed to continue nucleotide analogue therapy or decompensate cirrhosis as well as those persons coinfected with HIV or hepatitis C or with secondary renal diseases from other medical conditions such as diabetes or hypertension. The study's patients gave consent to participate with the longitudinal follow-up study. At baseline, at 3, 6, and 9 months, at and 1 year, fasting serum samples were collected for glucose, creatinine, electrolytes, phosphate, uric acid, and HBV viral load testing. Parathyroid hormone and vitamin D levels were assessed at baseline. Twenty-four hour urine samples were assessed for protein, creatinine, potassium, bicarbonate, phosphate, and uric acid. Random urine samples were evaluated with a dipstick and *β*2-microglobulin which is a specific marker of proteinuria of tubular origin [28]. Completed resolution of renal loss criteria was defined as completed reversal of PRTD, while partial resolution was called improvement of PRTD.

### 2.2. Laboratory Assays

The CKD-EPI equation was chosen to represent an estimated glomerular filtration rate (GFR). The equation was derived from GFR = 141 × min (creatinine/*κ*,1)^*α*^  ×  max (creatinine/*κ*,1)^−1.209^  ×  0.993^Age^  ×  1.018 [if female]. In this equation, *κ* is 0.7 for women and 0.9 for men, *α* is –0.329 for women and –0.411 for men, min indicates the minimum of creatinine/*κ* or 1, and max indicates the maximum of serum creatinine/*κ* or 1 [29]. Fractional excretion of potassium (FEK) ([urine potassium × plasma creatinine]/[urine creatinine × plasma potassium] × 100), fractional excretion of phosphate (FEPO_4_) ([urine phosphate × plasma creatinine]/[urine creatinine × plasma phosphate] × 100), and fractional excretion of uric acid (FEUA) ([urine uric acid × plasma creatinine]/[urine creatinine × plasma uric acid] × 100) were calculated from the serum and 24-hour urine samples [27, 30, 31]. This resulted in the tubular maximal reabsorption rate of phosphate to GFR (TmPO_4_/GFR) (plasma PO_4_ – ([urine phosphate × plasma creatinine]/urine creatinine)) (normal 2.8–4.4 mg/dL) [19].

The criteria for renal loss were defined as follows:

  Proteinuria = 24-hour urinary protein >150 mg.
  Glycosuria with normoglycemia = positive glucose dipstick (or urine glucose > 300 mg per day) while fasting glucose < 100 mg/dL.
  Phosphaturia = FEPO_4_ > 18% or 24-hour urine phosphate >1,200 mg.
  Uricosuria = FEUA > 15%.
  Renal potassium loss = hypokalemia with FEK > 6.5% or 24-hour urine potassium > 20 mEq per day.
  Renal tubular acidosis = serum bicarbonate < 19 mmol/L with normal gap acidosis.

### 2.3. Statistical Analysis

Data are expressed as mean ± standard deviation (SD) or median (interquartile range or IQR). Categorical and continuous variables between baseline and one year were compared with Chi-square and Wilcoxon's Signed Rank tests, respectively. The comparisons baseline data and the data at 3, 6, and 9 months and 1 year were done with multilevel mixed models. Random intercept and slope were included in the model to account for multiple measurements per individual. The effect of nucleotide analogue withdrawal on FEPO_4_ level was examined. Covariates considered in the different models included age, the presence of diabetes or hypertension, the duration of nucleotide analogue taking, and the type of PRTD at baseline. All analyses were adjusted for age and all other covariates associated with the outcomes in the multivariate model. A *P* value less than 0.05 was considered to be significant. Statistical analysis used SPSS version 16.0 (Chicago, IL) and Stata software version 14 (College Station, TX).

## 3. Results

### 3.1. Baseline Characteristic of CHB Patients with Nucleotide Analogue-Related PRTD

At the beginning of the study, 17 CHB patients whose nucleotide analogue therapy was discontinued were enrolled. At six months after stopping the nucleotide analogue therapy, one patient with a past history of LAM resistance developed a rebound in the HBV viral load while taking LAM monotherapy. Therefore, the TDF was added to LAM in this case, and he was excluded from further study. The remaining 16 patients were followed until the end of the study period. Baseline data are shown in Table 1. The median age [IQR] was 61.0 [59–66] years. Median [IQR] duration of nucleotide analogue therapy was 70.0 [49–75] months. Four patients had mild hypertension and diabetes mellitus without significant complications. Ten (62.5%) patients were treated with TDF-based regimen including TDF monotherapy or TDF add-on LAM. Six (37.5%) patients were on ADV monotherapy or ADV add-on LAM. Six patients in the TDF-based group had a history of previous ADV treatment. The workup for renal tubular function categorized the 16 patients into two groups according to the severity of PRTD: eight patients (50%) with subclinical PRTD and eight patients (50%) with overt PRTD.

A study of bone mineral density showed that ten patients (62.5%) had evidence of bone demineralization including three (18.8%) with osteoporosis and seven (43.8%) with osteopenia. The median [IQR] levels of vitamin D and parathyroid hormone were 28.7 [24.0–30.4] pg/mL and 34.8 [30.4–60.4] ng/mL, respectively. There was no difference in the median [IQR] levels of vitamin D and parathyroid hormone in patients with normal bone mineralization, osteopenia, and osteoporosis (29.3 [26.0–34.1], 28.9 [23.9–29.5], 26.5 [23.8–30.4] pg/mL (*P* = 0.51) and 39.9 [26.5–52.8], 30.1 [27.9–32.7], 44.8 [32.0–74.0] ng/mL (*P* = 0.20), resp.).

### 3.2. Improvement in GFR, Serum Phosphate, and Uric Acid and Reduction of Renal Loss of Phosphate, Uric Acid, 24-Hour Protein, and *β*2-Microglobulin after Nucleotide Analogue Withdrawal

**Figure 1:**
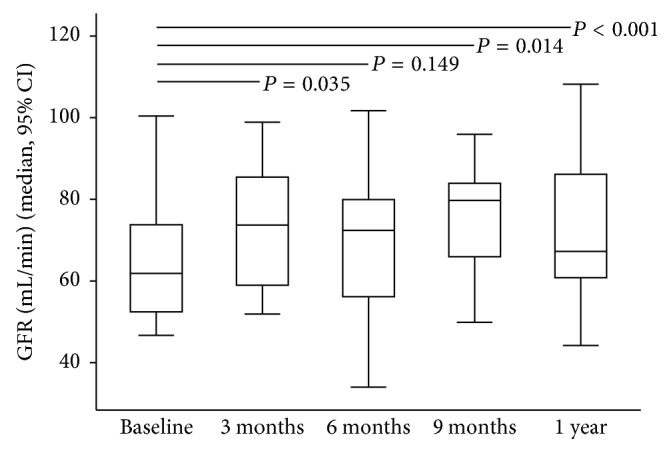
Increasing glomerular filtration rate (GFR) from baseline to one year after discontinuation of nucleotide analogue therapy.

**Figure 2:**
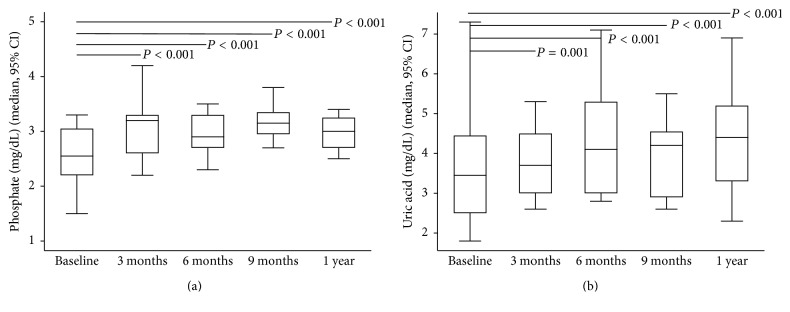
Rising serum phosphate and uric acid from baseline to one year after discontinuation of nucleotide analogue therapy. (a) Serum phosphate and (b) serum uric acid.

**Figure 3:**
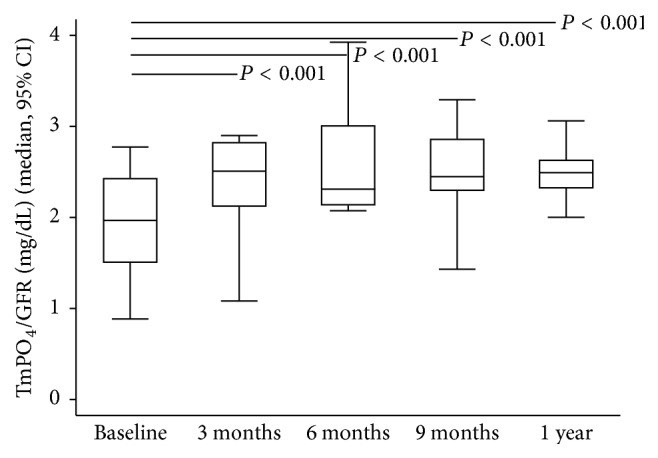
Increasing tubular maximal reabsorption rate of phosphate to GFR (TmPO_4_/GFR) from baseline to one year after discontinuation of nucleotide analogue therapy.

**Figure 4:**
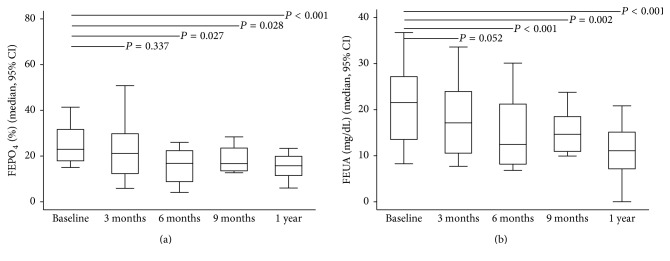
Reducing fractional excretion of phosphate (FEPO_4_) and uric acid (FEUA) from baseline to one year after discontinuation of nucleotide analogue therapy. (a) FEPO_4_ and (b) FEUA.

**Figure 5:**
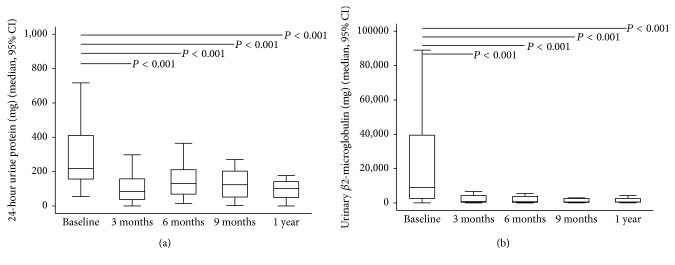
Decreasing 24-hour urinary protein (a) and urinary *β*2-microglobulin (b) from baseline to one year after discontinuation of nucleotide analogue therapy. (a) 24-hour urinary protein and (b) urinary *β*2-microglobulin.

After ADV or TDF was discontinued, antiviral therapy was switched to ETV and LAM monotherapy in nine (56.2%) and three (18.8%) patients, respectively. In addition, ADV or TDF was stopped in four (25%) patients with prior history of early add-on with LAM. The GFR increased significantly at 3, 6, and 9 months and one year after drug withdrawal while HBV viral load remained undetectable throughout the study period in the 16 patients (Figure 1). The significant and stepwise increase in the median levels of serum phosphate and uric acid occurred from three months to one year (Figures 2(a) and 2(b)). At the beginning of the study, oral phosphate supplementation was required in three patients. However, after six months, only one patient still required phosphate supplementation until the end of the study period. An increase in TmPO_4_/GFR at three months suggested that the improvements in renal tubular phosphate handling occurred soon after drug withdrawal (Figure 3); however, significant reductions in FEPO_4_ and FEUA were observed at six months (Figures 4(a) and 4(b)). The severity or type of PRTD at baseline was the only factor independently associated with FEPO_4_ (HR 2.78; 95% CI: 2.77–16.07, *P* < 0.005). In addition, the significant reduction in the renal losses of protein and *β*2-microglobulin occurred after nucleotide analogue therapy was withdrawn, and the phenomenon was noticed as early as three months after drug withdrawal (Figures 5(a) and 5(b)).

### 3.3. Clinical Outcomes of PRTD at One Year after Nucleotide Analogue Therapy Withdrawal

At one year after the discontinuation of nucleotide analogue therapy, the GFR, serum phosphate, uric acid, and tubular reabsorption of phosphate were significantly higher than the data at baseline (Table 2). Furthermore, serum creatinine and the renal losses of phosphate, uric acid, protein, and *β*2-microglobulin were significantly lower than the baseline data. The renal tubular dysfunction of all patients with subclinical PRTD was completely reversed one year after nucleotide analogue discontinuation. On the contrary, the renal tubular dysfunction of patients with overt PRTD was reversed in 5/8 (62.5%) of patients. The characteristic features of patients with complete reversal and incomplete reversal of PRTD are shown in Table 3. Improvements in tubular dysfunction were seen in all patients except for one with overt PRTD. PRTD persisted in this patient although nucleotide analogue was discontinued after more than one year of follow-up. The patient was a 59-year-old male with mild hypertension who had received nucleotide analogue therapy for 90 months (ADV for 62 months and followed with TDF for 28 months). However, five patients who received nucleotide analogue treatment for 91, 99, 101, 101, and 108 months had improvements in PRTD.

## 4. Discussion

This paper presents a longitudinal follow-up study after nucleotide analogue was withdrawn in 16 CHB patients who were affected by nucleotide analogue-related PRTD, mostly by switching to ETV and LAM. One case was excluded from the study at 6 months because of flare of LAM-resistant HBV while on LAM. HBV flare did not occur in the 16 CHB patients during the study period. The withdrawal of nucleotide analogue in CHB patient with LAM resistance carries a chance of the flare of drug-resistant HBV. This risk of HBV flare needs to be considered also with the risk of PRTD and bone demineralization. In this study, renal impairment and PRTD began to improve as early as three months after discontinuation of nucleotide analogue as seen from the rising TmPO_4_/GFR representing improved tubular reabsorption of phosphate and the declining 24-hour urinary protein and urinary *β*2-microglobulin, which is a 12 kDA tubular protein with a component of major histocompatibility complex class I molecule [32]. Urinary *β*2-microglobulin is known to be a specific marker of proximal renal tubular dysfunction, which has been studied in HIV patients who developed antiretroviral agents related to PRTD [28, 32–34]. The rapid decline of renal losses of urinary protein and *β*2-microglobulin after cessation of nucleotide analogue signified the restoration of proximal renal tubular structure and function. Previously, there have been a few case reports describing the reversal of ADV and TDF-related Fanconi's syndrome after TDF withdrawal in patients with CHB [12, 26]. In contrast, our study showed that nucleotide analogue-related PRTD could be resolved in 16 CHB patients after discontinuation of nucleotide analogue similar to the phenomenon that occurs in HIV-infected patients [21–25].

The complete reversal of nucleotide analogue PRTD occurred in 81% of the patients at one year. Only five (62.5%) patients with overt PRTD had complete reversal, contrasting with all patients with subclinical PRTD. Our study suggested that the chance of complete reversal of PRTD might depend upon the severity of the tubular dysfunction and the duration of the nucleotide analogue therapy, although our study was not adequately powered to confirm the assumption. We found only the association of the severity of RTD at baseline and the renal loss of phosphate. One year after discontinuation of nucleotide analogue therapy, overt PRTD still persisted in one patient with no significant medical comorbidities except for taking nucleotide analogues (ADV followed with TDF) for 90 months. However, PRTD improvements were seen in five patients treated with nucleotide analogues for longer than 90 months. Complete reversal occurred in four of the five patients. It is unclear why PRTD remained protracted in this case despite discontinuation of nucleotide analogues for more than one year. Rare variants in genes involved in the renal handling of nucleotide analogue may be associated with increased susceptibility to Fanconi's syndrome or overt PRTD [35]. Of note, the diagnosis of Fanconi's syndrome with a bicarbonate-loading test was not done to differentiate Fanconi's syndrome from overt RTD in this study. Our findings show that if PRTD was detected at an early (or subclinical) stage—the nucleotide analogue was discontinued in a timely manner—we may prevent the irreversible damage to the proximal renal tubular structure and its function.

Increased entry from the human organic anion transporter (hOAT) and decreased efflux into the tubular lumen and the direct mitochondrial toxicity are the proposed mechanisms of ADV-induced nephrotoxicity [36]. The mechanism of renal adverse effects on TDF is unclear. The accumulation of TDF within proximal renal tubules can lead to mitochondrial injury, which has been proposed as a mechanism of TDF-induced nephrotoxicity and PRTD [36]. Discontinuation of TDF helps prevent the progression of TDF-induced nephrotoxicity despite incomplete reversibility [21, 22, 36].

Long-term dysfunction of the proximal renal tubules leads to impaired tubular reabsorption of amino acids, glucose, bicarbonate, and phosphate [7–9, 11, 12]. Chronic phosphate depletion can cause hypophosphatemic osteomalacia, diffused bone pain, and bone fracture [7–9, 11, 12]. Prolonged hypophosphatemia with decreased renal phosphate absorption can lead to impaired bone health [37]. In this study, the prevalence of osteoporosis and osteopenia was 62.5%, which was close to the prevalence of nucleotide analogue-related bone demineralization reported from a previous study [38]. The long-term outcome of osteoporosis and osteopenia after nucleotide analogue therapy withdrawal is not known and should be further explored.

The strength of this study was an in-depth evaluation of proximal renal tubular function with serum, spot, and 24-hour urine samples periodically; however, the small cohort size is a limitation of this work. Although PRTD and nephrotoxicity can occur after long-term use of nucleotide analogue therapy, rapid detection of the problems and timely cessation of the nucleotide analogue can increase the chance of reversal and repaired defects of proximal tubular function. A comprehensive assessment of renal function, tubular reabsorption of phosphate, uric acid, tubular protein, and *β*2-microglobulin may be helpful in the early detection and discontinuation of nucleotide analogue therapy. However, one might argue that the cessation of ADV or TDF led to only laboratory improvement with statistical significance but lack of substantial clinical importance as in 7 patients who continued taking ADV or TDF and were not recruited to the study. In clinical practice, when TDF cannot be stopped in patients with PRTD detection, continuous monitoring of renal function, serum phosphate, uric acid, and urinary protein regularly should be carried out. If bone demineralization is detected, calcium and vitamin D should be supplemented. The next challenging task is to find new antiviral therapies that can replace ADV or TDF for long-term suppression of HBV replication with high efficacy without causing adverse effects of PRTD and nephrotoxicity. The benefit of tenofovir alafenamide for the reduction of nucleotide analogue-related nephrotoxicity remains to be proven [20, 39].

## 5. Conclusions

One year after withdrawal of nucleotide analogue use, PRTD was resolved in the majority of the CHB patients who were on long-term therapy of the drug. Changes in TmPO_4_/GFR, urinary protein, and *β*2-microglobulin raise the hypothesis that urinary biomarkers may represent an early indicator of PRTD recovery, although larger outcome studies are warranted to confirm these preliminary findings. The risk factors of permanent nucleotide analogue-related PRTD despite drug cessation require further study.

## Figures and Tables

**Table 1:** Baseline data of CHB patients with nucleotide analogue-related proximal renal tubular dysfunction (PRTD).

Characteristics	*N* = 16
Median age [IQR] (years)	61.0 [59–66]
Male, *n* (%)	8 (50)
Cirrhosis, *n* (%)	3 (18.8)
HBeAg positive, *n* (%)	6 (37.5)
Hypertension, *n* (%)	3 (18.8)
Diabetes, *n* (%)	1 (6.2)
Median duration of nucleotide analogue [IQR] (months)	70.0 [49–75]
Type of nucleotide analogue, *n* (%)	
(i) Tenofovir or tenofovir + lamivudine^*∗*^	10 (62.5)
(ii) Adefovir or adefovir + lamivudine	6 (37.5)
Severity of PRTD, *n* (%)	
(i) Subclinical	8 (50)
(ii) Overt	8 (50)

^*∗*^Six patients received adefovir. IQR, interquartile range.

**Table 2:** Renal function and proximal renal tubular dysfunction (PRTD) at baseline and one year after discontinuation of nucleotide analogue therapy.

	At baseline	At one year after drug discontinuation	*P* value
Serum creatinine (mg/dL)	1.1 [0.9–1.2]	1.0 [0.8–1.2]	0.023
GFR (mL/min)	61.9 [52.0–74.2]	67.3 [59.7–88.0]	0.032
Serum phosphate (mg/dL)	2.6 [2.2–3.1]	3.0 [2.7–3.3]	0.005
Serum uric acid (mg/dL)	3.5 [2.5–4.5]	4.4 [3.3–5.2]	0.002
TmPO_4_/GFR (mg/dL)	2.0 [1.5–2.4]	2.5 [2.3–2.6]	0.002
FE of phosphate (%)	22.9 [17.6–32.0]	15.8 [10.7–20.2]	0.005
FE of uric acid (%)	22.0 [15.3–30.0]	11.1 [7.1–15.2]	0.001
24 hour urinary protein (mg)	218.5 [153.5–414.8]	101.0 [48.0–144.0]	0.002
Urinary *β*2-microglobulin (mg/dL)	9,070.0 [1,655.0–41,025.0]	565.0 [252.5–2,790.0]	0.001

Data are expressed as median [interquartile range]; GFR, glomerular filtration rate; TmPO_4_/GFR, tubular maximal reabsorption rate of phosphate to GFR; FE, fractional excretion.

**Table 3:** Characteristics of patients with complete reversal versus incomplete reversal of proximal renal tubular dysfunction (PRTD) at one year.

	Complete reversal	Incomplete reversal	*P* value
Number	13	3	
PRTD staging at baseline, *n* (%)			0.10
(i) subclinical	8 (100)	0	
(ii) overt	5 (62.5)	3 (100)	
Age^*∗*^ (years)	62 [61–67]	59 [56–60]	0.14
Duration of nucleotide analogue^*∗*^ (months)	67 [47–91]	90 [77.5–95.5]	0.44
Diabetes, *n* (%)	1 (7.7)	0	0.81
Hypertension, *n* (%)	2 (15.4)	1 (33.3)	0.49

^*∗*^Data expressed as median [interquartile range].
